# Glaucoma awareness, knowledge, perception of risk and eye screening behaviour among residents of Abokobi, Ghana

**DOI:** 10.1186/s12886-016-0376-0

**Published:** 2016-11-17

**Authors:** Virtue Fiawokome De-Gaulle, Phyllis Dako-Gyeke

**Affiliations:** Department of Social and Behavioral Sciences, School of Public Health, College of Health Sciences, University of Ghana, P. O. Box LG 13, Legon, Accra, Ghana

**Keywords:** Glaucoma, Knowledge, Perception, Eye screening, Abokobi, Ghana

## Abstract

**Background:**

Although glaucoma is the lead cause of irreversible blindness globally, the condition shows no signs or symptoms until later stages. Knowledge about the disease is known to influence utilization of eye screening services. This study aimed at understanding knowledge and perception of risk for glaucoma, as well as eye screening behaviour among residents of Abokobi, a peri-urban community.

**Methods:**

This was a cross-sectional study that employed quantitative data collection methods, with the use of a questionnaire. Descriptive statistics were used to describe the socio-demographic characteristics, knowledge about glaucoma and eye screening behaviour. Also, associations between socio-demographic factors and awareness as well as perception of risk were analysed using Chi-square test or Univariate Fisher’s exact test.

**Results:**

Out of a total of 300 respondents, 60.3 % were females and 39.3 % were aware of glaucoma. Majority (99.1 %) of respondents aware of glaucoma also agreed the disease can result in blindness with only (28 %) affirming that blindness from glaucoma is irreversible. Nearly half (49.7 %) of the respondents perceived themselves to be at risk of developing glaucoma. The results showed that age and education (*p* <0.0001) were statistically significant with glaucoma awareness. Approximately, 20.7 % of the respondents have had their eye screened with just a few (4.3 %) screening for glaucoma.

**Conclusion:**

Although glaucoma awareness was high, the findings display inadequate knowledge about glaucoma. There is a need to effectively inform and educate people about the disease.

**Electronic supplementary material:**

The online version of this article (doi:10.1186/s12886-016-0376-0) contains supplementary material, which is available to authorized users.

## Background

Glaucoma is defined as a neuropathy with structural (optic disc damage) and functional loss (visual field loss) [1, 2]. Glaucoma causes irreversible defects in the visual field and can lead to total blindness when left untreated [3]. Traditionally, glaucoma is classified broadly into primary and secondary glaucoma, with angle closure glaucoma (ACG) and open angle glaucoma (OAG) being the most common types [1, 3, 4]. Angle closure glaucoma is known to be highly prevalent among East Asians [5] whilst Africans and people of African descent record higher prevalence of open angle glaucoma [6].

Glaucoma is a global public health problem, because it is the highest cause of irreversible blindness and the second leading cause of blindness after cataract [2, 4, 6]. Yet, blindness is not always highlighted by health and public health practices, particularly when mortality indicators are used [7]. Quigley reports that glaucoma is undiagnosed in nine out of ten affected people globally [37]. Past trends in the disease prevalence reveal that between the year 2000 and 2013 over 60 million people in the world had glaucoma [1, 2, 9, 10]. The prevalence of the disease is however projected to further increase as the global population increases in both age and number [11]. Future projections of glaucoma cases reveal that about 76 to 80 million people will develop the disease by the year 2020 [1, 9]. This will further upsurge to about 118.8 million people by the year 2040 globally [8]. Africa is estimated to have the highest glaucoma prevalence of 4.79 as compared to 2.93 in Europe and 3.40 in Asia [9]. Unfortunately, it is one of the neglected diseases and the second highest cause of blindness after cataract in the region [12]. Cook stated that West Africa could have higher glaucoma prevalence than East and Southern Africa [13]. Hence, it is not surprising that three population based prevalence studies conducted in Ghana among various populations, in West Africa indicated a high glaucoma prevalence of 6.5 % in Tema, [14] 7.6 % among adults in the Volta region [15] and 8.5 % in the Akuapim South district [16]. Whereas, two population based studies in Tanzania an east African country and South Africa revealed a prevalence of 4.16 and 4.5 % respectively [17, 18].

High glaucoma morbidity among some African communities may be attributed to low awareness, under-utilization of eye care services as well as the limited availability of treatment procedures [12, 19–21]. Several studies have reported low levels of glaucoma awareness [19–24]. Unfortunately, there are limited efforts directed at awareness creation of glaucoma in Africa [12, 25]. Awareness of the disease can influence regular eye checks for early detection and prognosis of this asymptomatic disease [15, 26]. A lot of people in developing countries do not have regular and timely eye examinations due to lack of awareness or knowledge about glaucoma [21]. Because there are also no overt symptoms or associated pain, people do not undergo screening, a requirement for early diagnosis [12]. This results in a high proportion of patients presenting advanced stages of the disease where there is loss of sight in an eye or both eyes [25] Most Africans, present either unilateral or bilateral blindness of glaucoma due to late detection of the disease [25, 27]. Furthermore, African countries are highly underserved in terms of eye care services, with about a million people to one ophthalmologist [12, 28, 29].

In Ghana the healthcare system does not currently provide enough incentive for people to seek regular ophthalmologic care [30]. Rather, the Ghana Health Service prioritises dealing with infectious diseases caused by unsanitary conditions neglecting glaucoma and other eye diseases [30]. Ghanaians are generally not in the habit of seeking medical care unless there is a foreseeable problem [30]. For a developing country like Ghana, where people are struggling to meet their basic needs, it is a luxury to take time and money to screen, much more treat a condition like glaucoma that is not yet causing visual loss [15]. A population-based survey conducted in the Cape Coast Metropolis of Ghana reported poor eye health seeking behaviour [31]. Respondents preferred alternative eye care services such as traditional healers and local pharmaceutical shops than seeing an ophthalmologist or optometrist [31].

Use of alternative healthcare services is driven by how glaucoma is often perceived [32, 33]. Whereas some perceive it as a consequence of old age [32] others see blindness as a condition caused by evil spirits or enemy scheming [33]. This situation, coupled with poverty and inadequate knowledge serves as a key barrier limiting access to eye care services in Africa [34]. To better understand factors leading to such perceptions, this study seeks to assess awareness, perception of risk of glaucoma and eye screening behaviour in a peri-urban population with limited access to eye care. A better understanding of this context can lead to interventions that encourage utilization of eye screening services to enhance early diagnosis and treatment of individuals with glaucoma in a country like Ghana with a high prevalence.

## Methods

This is a cross-sectional survey conducted in Abokobi, the district capital of the Ga East Municipality. Ga East is one of the districts in Greater Accra region with a population of about 33,500 people. Eligibility criteria for the study population was based on age and residence of the individual. The study population is adults, 18 years and above who are residents of Abokobi.

### Sampling procedure

Simple random sampling was done to select study population. The sample size of 300 was determined using Cochran’s formula (1963–1975). The principal investigator together with the research assistants drove through the community and counted the residential structures in it. Approximately 2700 structures were counted and this was divided by the sample size of 300 to get an interval of nine. In the community, the principal investigator and research assistants tossed a bottle to determine a random direction. The first structure in the determined direction was used as the starting point and an interval of nine structures was counted to determine the next structure to be sampled until the sample size of 300 was obtained. Eligible respondents found at home or workplace were asked to ballot and either pick a yes or no after which the respondent who picked the yes was asked willingness to participate and consent taken. One respondent was taken from each structure.. Ethical approval for the study was obtained from the Ghana Health Service. Permission was also obtained from the Health Directorate of the Ga East District.

### Data collection and analysis

Interviewer administered structured questionnaires was used to obtain data from the respondents. Data was collected by the principal investigator and two research assistants who were trained on research ethics, seeking informed consent and the research questionnaire (Additional file 1).

Data collection involved face-to-face interviews with respondents. The local dialects Ga or Twi were mainly used for data collection. Averagely about 20 min was used to answer a questionnaire (Additional file 2). The questionnaire was designed to assess glaucoma awareness, knowledge, perception of risk of glaucoma and eye screening behaviour. Information on socio-demographic characteristics of respondents was also collected.

The SPSS program, version 20.0 was used for data entry, cleaning and analysis. Descriptive statistics, including frequencies and percentages were used to describe the socio-demographic characteristics, glaucoma knowledge and eye screening behaviour of respondents. The association between awareness of glaucoma, perception of risk and socio-demographic factors such as age, sex, marital status, religion, educational level and ethnicity, was analysed using the Chi-square test or Univariate Fisher’s exact test. A two-tailed *p*-value of less than 0.05 was considered statistically significant.

## Results

Three hundred respondents were interviewed during the study period. The age of the respondents ranged between 18 and 70 years with majority (35.7 %) of the respondents being between 40 and 49 years old (Table 1). Most (60.3 %) of them were females, with about 67.7 % of the respondents being married. The respondents were predominantly Christians (93 %) with Muslims making up 7 %. Majority (34.7 %) of the respondents had completed Junior High School with traders forming about 35.3 % followed by artisans (26.7 %). Most (43.0 %) of the respondents were Ga/Dangme’s, followed by Akan’s (25.3 %) and Ewe’s 15.7 %.

**Table 1:** Socio-demographic characteristics of respondents (*N* = 300)

Characteristics	Frequency (n)	Percentage (%)
Age group (Years)		
18–29	39	13.0
30–39	64	21.3
40–49	107	35.7
50–59	43	14.3
60–69	36	12.0
70+	11	3.7
Sex		
Male	119	39.7
Female	181	60.3
Marital status		
Married	203	67.7
Divorced	16	5.3
Widowed	33	11.0
Single	48	16.0
Religion		
Christian	279	93.0
Muslim	21	7.0
Educational level		
None	94	31.3
Primary	69	23.0
Junior High School	104	34.7
Senior High/ Vocational	30	10.0
Tertiary	3	1.0
Occupation		
Unemployed	46	15.3
Civil servant	10	3.3
Private company worker	56	18.7
Trader	106	35.3
Artisan	80	26.7
Student	5	1.7
Ethnicity		
Akan	76	25.3
Ga/Adangme	129	43.0
Ewe	47	15.7
Guan	19	6.3
Mole-Dagbani	21	7.0
Grussi	8	2.7

### Socio-demographic characteristics by glaucoma awareness and source

Thirty nine percent (*n* = 118) of all the respondents indicated they had heard about the eye condition glaucoma. Out of the respondents who had heard about glaucoma, 56.8 % of them were females (Table 2). Although a higher proportion of female respondents were observed to have heard about glaucoma, the awareness of glaucoma in relation to sex was not statistically significant (*x*2 = 1.026; df = 1; *P* = 0.31). Likewise religious affiliation was also not statistically significant (*x*2 = 0.118; df = 1 *P* = 0.732) irrespective of the fact that a greater percentage of Christians (36.3 %) affirmed to glaucoma awareness as opposed to 3.0 % of Muslims. However, there was a significant relationship between glaucoma awareness and age (*x*2 = 31.582; df = 5 *p* < 0.0001) with most (42) of the respondents between the ages of 40 and 49 years being aware of the disease. Likewise, most of the Junior High School graduates were aware of glaucoma and this was also statistically significant (*x*2 = 20.372; df = 4 *p* < 0.0001).

**Table 2:** Socio-demographic characteristics by Glaucoma Awareness and Source (*N* = 118)

Characteristics	Awareness (n)	*x* ^2^	*P* – value	Percentage %
Sex				
Male	51	1.03	0.31	43.2
Female	67			56.8
Age				
18–29	8			6.8
30–39	22			18.6
40–49	42	31.58	*p* < 0.0001	35.6
50–59	20			16.9
60–69	10			22.0
Total	118			100
Religious affiliation				
Christian	109			92.4
Muslim	9	0.12	0.73	7.6
Total	118			100
Educational status				
None	21			17.8
Primary	35			29.7
Junior High School	50	20.37	*p* < 0.0001	42.4
Senior High School	10			8.5
Tertiary level	2			1.7
Total	118			100

### Respondents knowledge on glaucoma (*N* = 118)

A three point scale of agree, disagree and do not know was used to ask respondents who said they were aware of glaucoma a number of knowledge statements about the disease. These responses were used to assess their knowledge of the disease. Most (99.1 %) of the respondents agreed that glaucoma results in blindness. About 77.1 % of the respondents also agreed that glaucoma causes reduction of visual acuity. Approximately 64.4 % of them also affirmed it was possible to have glaucoma without knowing with 28.8 % of the respondents agreeing that blindness from glaucoma is irreversible and the least (4.2 %) number of respondents agreeing that Africans stand a higher risk of developing the disease than whites (Table 3).

**Table 3:** Respondents knowledge on glaucoma (*N* = 118)

Knowledge statements	Frequency (n)	Percentage (%)
Glaucoma results in blindness	117	99.1
Glaucoma causes reduction of visual acuity	91	77.1
It is possible to have glaucoma without knowing it	76	64.4
Blindness from glaucoma is irreversible	34	28.8
Africans develop glaucoma more than whites	5	4.2

### Perception of risk of glaucoma (*N* = 149)

Generally all respondents were asked if they perceived themselves to be at risk of developing glaucoma of which 149 out of the 300 respondents perceived themselves to be at risk of developing the disease. Respondents were further asked if they perceived their sex, age, religion, occupation, educational or marital status made them susceptible to developing glaucoma. The perception of risk of glaucoma in relation to sex, age and religion was not statistically significant (Table 4). However, glaucoma perception of risk was statistically significant with occupation, education, marital status with respondents who had completed Junior High School, traders and married ones perceiving themselves to be at risk of developing the disease.

**Table 4:** Perception of risk of glaucoma (*N* = 149)

Factors * Perception of risk of glaucoma	*x* ^2^	Df	*P* - value
Sex	1.94	1	.16
Age	7.440	5	.190
Religion	0.419	1	.518
Occupation	11.915	5	.03
Education	29.981	4	˂0.0001
Marital status	41.293	4	˂0.0001

### Eye screening of respondents (*N* = 62)

Out of the 300 respondents, only 20. 7 % (*N* = 62) had ever gone for an eye screening. Respondents reported screening for eye conditions such as glaucoma, refractive error, pain in eyes and even driver’s license (Table 5). Glaucoma was the least (13) eye condition screened for whereas screening for driver’s license placed highest (18). Half of the respondents who had ever had an eye screening went voluntarily with just a few (13) being as a result of referrals. Most (45) of the respondents screened for their eyes in either a private hospitals or clinic, followed by government facilities (16) with outreaches being the least (1) place for eye screening.

**Table 5:** eye screening of respondents (*N* = 62)

	<6 months	12–24 months	>36 months	Total
Eye condition screened for				
Glaucoma	13	0	0	13
Refractive error	1	12	3	16
Pain in eye	0	7	8	15
Driver’s license	4	10	4	18
Total	18	29	15	62
Reasons for eye screening				
Voluntary	1	19	11	31
Referral	13	0	0	13
Driver’s license requirement	4	10	4	18
Total	18	29	15	62
Place of eye screening				
Gov’t Hosp./Clinic	10	3	3	16
Outreach	0	0	1	1
Private Hosp./Clinic	8	26	11	45
Total	18	29	15	62

## Discussion

This study investigated awareness, knowledge and perception of risk of glaucoma among adults in a peri-urban population in Abokobi, the district capital of Ga East Municipality. Glaucoma is the world’s leading cause of irreversible blindness [35]. In Sub-Saharan Africa, glaucoma is one of the neglected diseases and the second highest cause of blindness after cataract [12] Glaucoma is thus a global public health problem, in 2013, the estimated prevalence of glaucoma worldwide was 64.3 million people between the ages of 40 and 80 years [9]. Africa recorded the second highest number of cases of 8.3 million people [9].

The findings from this study revealed minimal level (39.3 %) of awareness aand basic knowledge of glaucoma as well as low perception of risk among respondents. The low awareness of the disease is not surprising as a study conducted by Ntim-Amponsah et al. in Ghana showed that about 94 % of people diagnosed with glaucoma were unaware that they had the disease prior to being diagnosed. Furthermore, the Low awareness levels and knowledge of glaucoma in this study population could be due to the little or non-existent publicity about glaucoma in the community or the country as a whole. Moreover, access to ophthalmic care in Abokobi may have contributed to the low levels of glaucoma awareness since Abokobi’s ophthalmic needs are attended to by one ophthalmic nurse who visits the Abokobi Health Centre once a week. Adequate access to ophthalmic services and proper utilisation of eye care services can create greater awareness and publicity to information about various eye diseases including glaucoma [36] Unfortunately many rural towns do not have an ophthalmologist in practice [37]. Other studies in some African countries have reported low level of awareness of glaucoma [19–21, 24]. In South-western Ethiopia, a very low (2.4 %) glaucoma awareness was reported in participants who partook in an ophthalmic outreach programme [19]. A much lower (0.32 %) level of glaucoma awareness was reported in a rural population based study in Andhra Pradesh, India and this was attributed to limited access to medical and diagnostic care in that community [38]. These findings show that glaucoma awareness is generally low irrespective of the geographical location. More astonishing is the finding of very poor awareness among non- ophthalmic doctors, and non-medical staff in a facility based study in Nigeria [21]. Contrastingly, as far back as 1995, Livingston et al. reported 70 % level of awareness in a population based sample study [39] in Australia. Similarly, in Canada, a 41.2 % of glaucoma awareness level was reported in a cross sectional facility based survey [22]. The awareness levels of glaucoma in these industrialized countries are higher as compared to African countries. These findings further augment the fact that European countries have more established eye care systems than in African countries. Glaucoma screening is free for people with a family history of the disease in the United Kingdom [40]. Kingman stated that there is one or less ophthalmologist for every million African, whereas there is one for every10,000 people in Europe [29]. Egbert also reported the ophthalmologist to patient ratio to be 1: 1000 in Ghana. [12]. This is an indication that the human resources needed to satisfy global eye care is limited but worse in Africa with Ghana not being an exception.

Age was statistically significant with glaucoma awareness in this study, respondents between the ages of 40 and 49 years were significantly more aware of glaucoma than the other age brackets. The low level of glaucoma awareness (2.7 %) in the younger generation could be because younger people perceive eye diseases to be a problem of the older generation. They therefore do not pay much attention to eye diseases, much more a disease like glaucoma which is asymptomatic. A study in the United Kingdom among African Caribbean’s on glaucoma awareness and perception of risk discovered that they perceived themselves to be at risk of glaucoma in their old age [32]. However, respondents over 70 years who are most at risk of developing glaucoma were the least aware in this study. Education was also significantly associated with glaucoma awareness, respondents who had completed the junior high school ranked highest among those aware of glaucoma. Similar findings were reported in South-Western Ethiopia where glaucoma awareness was significantly associated with attaining high school education or better in an outreach ophthalmic programme [19]. Although the association between glaucoma awareness and gender was not statistically significant in this study, females were 1.3 times (22.3 %) more likely to be aware of glaucoma than males (17.0 %). This could be as a result of the gender roles assigned to women as caregivers of the sick and disabled in our homes and society. Since glaucoma is usually noticed during the advanced stages in most patients in Ghana where there is considerable loss of vision [27], women are more likely to escort these patients to the hospital as well as care for them, and they are therefore more likely to gain knowledge about the disease during their visits to the health facilities. In the same way, females were found to be significantly more aware (59 %) of glaucoma in an Australian study than males [40]. Krishnaiah et al., conversely, reported the opposite of our findings, with females being less significantly aware of glaucoma than men [38]. Generally, the respondents who were aware of glaucoma in this study had fair knowledge about the disease, because 94.7 % agreed that glaucoma results in blindness and 79.7 % also said it causes reduction in visual acuity. It is likely to say or agree that a disease that affects the eye leads to reduction in visual acuity as well as results in blindness. However, only 26.3 % of them agreed blindness from glaucoma is irreversible, showing that only a few had correct knowledge about the disease. This is in concert with the findings of Krishnaiah et al., where majority of the respondents who were aware of glaucoma did not know visual loss due to glaucoma was permanent or irreversible [38]. This study conceptualized that demographic characteristics such as sex, age, education and religion would influence the perception of risk of glaucoma. The results revealed that education and marital status were significantly associated with perception of risk of glaucoma. Married respondents and those who have attained Junior High School perceived themselves to be at risk. Sex, age and religion were not significantly associated with perception of risk of glaucoma as conceptualized. It is very likely people will perceive themselves not to be at risk of a disease like glaucoma that shows no symptoms for a long time. Awareness of these risk factors will prompt some people to seek eye care for early detection and prognosis. The perception that visual loss is a normal consequence of ageing could also be the reason for low perception of risk of the disease in this study population of mostly middle aged and young adults. Therespondents who perceived themselves not to be at risk attributed it to being protected by their religious faith as Christians. A community-based study in Anambra State of Nigeria documented that people generally perceive eye diseases and blindness as being caused by evil spirits or enemy machination [33]. This could explain why the respondents in this current study had low perception of risk because of divine protection from their religious faith probably against evil spirits or the enemy machination. Most (79.3 %) of the respondents have never undergone eye screening although they complained of various eye problems such as itchy, teary, and blurry vision. Financial reasons could account for this, in a developing country like Ghana where people are probably struggling to meet their daily needs; they would find it as an unreasonable luxury to take time and money to go for early screening to treat a condition that is not yet causing visual loss. Additionally, the limited availability of ophthalmic services in the community could be a reason because Abokobi’s ophthalmic needs are attended to by a visiting ophthalmic nurse once a week. Bowen, (2011) found that medical care did not exist in most communities in Ghana. Health education and presence of ophthalmological care in underprivileged communities will improve the level of awareness of eye diseases [41].

### Limitation of the study

The translation of some English words to the local dialects Ga and Twi may have resulted in some missing emphasis or misrepresentation on the translated words.

## Conclusion

The findings of this study on glaucoma awareness and knowledge bring to light the need to intensify efforts to create awareness and reduce morbidity from this disease. Health workers must actively take up mass education campaigns to clarify misconceptions about the disease and its risk factors.

## Additional files

Additional file 1: Questionnaire on glaucoma awareness, knowledge. (DOCX 75 kb)
Additional file 2: Questionnaire validation procedure on glaucoma awareness. (DOCX 12 kb)
