# Supplementary material for: Glaucoma awareness, knowledge, perception of risk and eye screening behaviour among residents of Abokobi, Ghana
Source: BMC Ophthalmol. 2016 Nov 17;16:204. doi: 10.1186/s12886-016-0376-0 (PMC5114832; doi:10.1186/s12886-016-0376-0)
Supplement: Additional file 1: — Questionnaire on glaucoma awareness, knowledge. (DOCX 75 kb) [file 12886_2016_376_MOESM1_ESM.docx]

**Consent Form**

**Study title:** **Glaucoma awareness, knowledge, perception of risk and eye screening behaviour among residents of Abokobi, Ghana**

**Institutional Affiliation:** School of Public Health**,** College of Health Sciences**,** University of Ghana, Legon.

**Background**

The principal investigator is Virtue Fiawokome De-Gaulle, currently a master’s student of the School of Public Health, Legon. She is undertaking a study on the factors influencing perceived risk to glaucoma in Abokobi. This study is to find out the level of knowledge and awareness of glaucoma in the study population. Furthermore, the study aims at identifying the factors that influence the perceived risk of research participants to glaucoma as well as their eye screening behaviour. The study is for academic purposes and a requirement for the award of Master of Science Degree in Applied Health Social Science under the supervision of Dr Phyllis Dako-Gyeke of the School of Public Health, University of Ghana, Legon.

**Procedure**

Face to face interviews will be conducted using structured questionnaires

**Risks and Benefits of the study:**

There is no risk associated with the research protocol. There is no foreseeable harm that may arise from participating in the research. The research will be beneficial because it will add up to literature on glaucoma studies in Ghana.

**Confidentiality and Anonymity** Names will not be included in the demographic data to be collected from research participants ensuring anonymity. Information collected will not be shared with third parties not directly involved in the study. This research is purely for academic purposes.

**Before taking consent**

If you have any question, be at ease to ask. If you have questions you wish to ask later, or clarification on issues regarding the research, please do not hesitate to contact the principal investigator (Virtue Fiawokome De-Gaulle) on 0261668108 or email **vdegorl@yahoo.com**. You can also contact the Academic supervisor Dr. Phyllis Dako-Gyeke on [**gyekenay@yahoo.com**](mailto:gyekenay@yahoo.com) **or the Ethical Review Committee Administrator Hannah Frimpong on 0243235225**.

**PARTICIPANT**

I ………………………………………………………………………………….. Having been adequately informed about the purpose, procedure, potential risks and benefits of this study, I have had the opportunity to ask questions which has been answered to my satisfaction. I know I have the right to redraw from the study without any loss or omitted from any benefits due me. Having gone through the consent form thoroughly, I agree to enrol in this study.

Name of Participant……………………………………………………………………………

Signature or thumb print: ……………………………………………………..

Date ………………………..

**Interviewer’s statement:**

I have explained the procedure to be followed in this study to the participant in the language that he or she understands best and he or she has agreed to participate in the study.

Signature of interviewer……………………………… Date ………………………

**Questionnaire on glaucoma awareness, knowledge, perception of risk and eye screening behaviour among residents of Abokobi, Ghana**

Hello, my name is _______________________________________ and I am a student of the School of Public Health at the University of Ghana, Legon. I am conducting a research on factors influencing perceived risk of glaucoma in Abokobi. I would very much appreciate your participation in this research. Be rest assured that all of the answers you give will be confidential. Participation in this research is completely voluntary. However, I hope you will participate since your views are important.

**Section A: Background characteristics**

| 1. Sex: [0] Female [1] Male 2. Age: [0] 18-29 [1] 30-39 [2] 40-49 [3] 50-59 [4] 60-69 [5] 70+ 3. Highest level of education:   [0] None [1] Primary [2] Junior High [3] Senior High [4] Tertiary  Marital status:  [0] Single [1] Cohabiting [2] Married [3] Divorced/Separated [4] Widowed   1. Religion:   [0] None [1] Christian [2] Muslim [3] Traditional African  Other:   1. Ethnicity: [0] Akan [1] Ga/Dangbe [2] Ewe [3] Guan [4] Mole Dagbani [5] Grussi   Other:   1. Current employment status: [0] Unemployed [1] Employed   Occupation: [0] Civil servant [1] Private company worker [2] Trader [3] Artisan [4] Farmer [5] Student   1. Area of residence: |
| --- |

**Section B: Awareness of eye conditions**

| 1. Are you familiar with any of the following eye diseases?   [0] None [1] Cataract [2] Glaucoma [3] Onchocerciasis [4] Trachoma [5] Refractive error   1. Have you ever heard of an illness called glaucoma?   [0] No [1] Yes [2] Don’t know |
| --- |
| 1. If yes, where did you hear it from?   [1] Mass media [2] Relative [3] Friend [4] Health worker Other: |

**Section C: Knowledge of glaucoma**

| Respondent’s knowledge on glaucoma. 1= Agree 2=. Disagree 3=.Do not know | | 1 | 2 | 3 |
| --- | --- | --- | --- | --- |
|  | Glaucoma results in blindness |  |  |  |
|  | Looking directly at the sun causes glaucoma |  |  |  |
|  | Blindness from glaucoma is not curable |  |  |  |
|  | Glaucoma leads to reduction of how clearly one can see |  |  |  |
|  | Signs and symptoms of glaucoma are itchy and swollen eyes |  |  |  |
|  | The risk of developing glaucoma increases with age |  |  |  |
|  | Glaucoma limits the ability to see a wider spatial environment |  |  |  |
|  | Glaucoma can never be detected unless blindness occurs |  |  |  |
|  | It is possible to have glaucoma without knowing it |  |  |  |
|  | Blindness from glaucoma is curable |  |  |  |
|  | African’s stand a higher chance of developing glaucoma than Whites. |  |  |  |
|  | The chances of developing glaucoma is high if a family member has it/ Glaucoma could be hereditary |  |  |  |

**Section D: Perception of risk of glaucoma**

| 1. Do you think you stand the risk of developing glaucoma?   [0] No [1] Yes [2] Don’t know  If yes why?  [1] Existing family history [2] Have sight/eye problems [3] Everyone is susceptible  Other:   1. If no why? [1] No family history [2] No sight/eye problems [3] religious reasons (protection) Other:   Please, I would like to know if any of the factors listed below apply to you. Please indicate if you think the factors that apply to you make you susceptible to developing glaucoma.   \|  \| Factors \| Please tick if applicable to you  1= Yes 2= No 3= Don’t know (DK) \| Do any of these factors influence your risk of developing glaucoma? \| \| --- \| --- \| --- \| --- \| \|  \| Age  (18 to 34 years)  (35 years and above) \| Yes  No  Dk \| Yes  No  Dk \| \|  \| Sex (Male / Female) \| Yes  Dk  No \| Yes  No  Dk \| \|  \| Type of job (smoke, dust, computer, saw mill) \| Yes  No  Dk \| Yes  No  Dk \| \|  \| Race (African) \| Yes  No  Dk \| Yes  No  Dk \| \|  \| Family history of glaucoma \| Yes  No  Dk \| Yes  No  Dk \| \|  \| Diabetes mellitus \| Yes  No  Dk \| Dk  No  Yes \| \|  \| Trauma to eyes \| Yes  No  Dk \| Yes  No  Dk \| \|  \| Use of cosmetics and makeup \| Yes  No  DK \| DK  No  Yes \| \|  \| Hypertension \| Yes  No  Dk \| Yes  No  Dk \| |
| --- | --- | --- | --- | --- | --- | --- | --- | --- | --- | --- | --- | --- | --- | --- | --- | --- | --- | --- | --- | --- | --- | --- | --- | --- | --- | --- | --- | --- | --- | --- | --- | --- | --- | --- | --- | --- | --- | --- | --- | --- |

**Section D: Perception of risk of glaucoma**

| 1. Have you ever gone for an eye screening? [0] No [1] Yes 2. If yes, what eye condition were you screened for? [1] Cataract [2] Glaucoma [3] Onchocerciasis [4] Trachoma [5] Refractive error 3. What was the reason for undergoing the eye screening? [1] No reason [2] Voluntary reasons [3] Hospital protocol [4] Referral Other: 4. When was the last time you went for an eye screening? [1] Within 6 months [2] Within 12 months [3] Within 24 months [4] Over 36 months 5. Where was the screening done? [1] Government Hospt./Clinic [2] Private Hospt./Clinic [3] Outreach programme 6. If no, Why? [0] No reason [1] No sight/eye problems [2] No money [3] No known eye centre Other: 7. Have you ever been screened for glaucoma? [0] No [1] Yes [2] Don’t know 8. Where was the screening done? [1] Government Hospt./Clinic [2] Private Hospt./Clinic [3] Outreach programme 9. Have you ever heard of any health outreach programme in your community?   [0] No [1] Yes [2] Don’t remember  If yes have you ever attended one? [0] No [1] Yes   1. Would you attend any future health outreach programme on eye screening? [0] No [1] Yes [2] Not sure 2. Do you think you have any eye problem? [0] No [1] Yes 3. If yes, describe it. [1] Itchy eyes [2] Pain in eyes [3] blurry vision [4] Refractive error Other: |
| --- |
