# Supplementary material for: Glaucoma awareness, knowledge, perception of risk and eye screening behaviour among residents of Abokobi, Ghana
Source: BMC Ophthalmol. 2016 Nov 17;16:204. doi: 10.1186/s12886-016-0376-0 (PMC5114832; doi:10.1186/s12886-016-0376-0)
Supplement: Additional file 2: — Questionnaire validation procedure on glaucoma awareness. (DOCX 12 kb) [file 12886_2016_376_MOESM2_ESM.docx]

**QUESTIONNAIRE VALIDATION PROCEDURE**

Reading of published literature on glaucoma in Ghana revealed that not much has been done on glaucoma awareness, knowledge and eyes screening behaviour, therefore this study was born out of the need to fill that research gap. Using the research questions and objectives of the study, a number of questions were generated to assess glaucoma awareness, knowledge, perception of risk and eye screening behaviour among residents in Abokobi, a peri urban community with limited eye care facilities. The sample size of the study was calculated using Cochran’s formula (1963-1975). To ensure content validity, the draft questionnaire was shown to an optometrist and ophthalmologist to ascertain whether the questions being asked were relevant in answering the research questions and addressing the objectives of the study. Their comments and feedback were used in revising the questionnaire. The entire research protocol was reviewed by a panel of research experts of the Ghana Health Service Ethical Review Committee and comments on the questionnaire was addressed accordingly. The questionnaire was pilot tested on adults 18 years and above in a peri urban community that had similar characteristics as the study area. Data collected during the pilot test was analysed using SPSS. The questionnaire was revised after the pilot test and the final questionnaire was printed for data collection.
